# Development of an Automated Crack Detection System for Port Quay Walls Using a Small General-Purpose Drone and Orthophotos

**DOI:** 10.3390/s25144325

**Published:** 2025-07-10

**Authors:** Daiki Komi, Daisuke Yoshida, Tomohito Kameyama

**Affiliations:** Graduate School of Informatics, Osaka Metropolitan University, Osaka 558-8585, Japan

**Keywords:** orthophoto, port quay wall, crack detection, deep learning, small general-purpose drone

## Abstract

**Highlights:**

**What are the main findings?**

- A cost-effective system using YOLOR and a novel image processing method (overlapping tiling and pseudo-altitude slicing) achieves reliable 1 mm crack detection, a capability crucial for proactive maintenance.
- In real-world evaluations, combining public and original datasets proved effective, and an *F*_1_ score-based ablation study revealed a key trade-off in the system’s performance. The system can be configured to either maximize *Recall* or achieve a best-balanced performance that is comparable to that of a commercial system.

**What are the implications of the main findings?**

- The system provides an accessible, affordable crack inspection tool for local governments, enabling more frequent and systematic monitoring of port infrastructure with limited resources.
- The system’s use of georeferenced orthophotos supports advanced, data-driven asset management, improving long-term maintenance planning and quantitative deterioration tracking.

**Abstract:**

Aging port infrastructure demands frequent and reliable inspections, yet the existing automated systems often require expensive industrial drones, posing significant adoption barriers for local governments with limited resources. To address this challenge, this study develops a low-cost, automated crack detection system for port quay walls utilizing orthophotos generated from a small general-purpose drone. The system employs the YOLOR (You Only Learn One Representation) object detection algorithm, enhanced by two novel image processing techniques—overlapping tiling and pseudo-altitude slicing—to overcome the resolution limitations of low-cost cameras. While official guidelines for port facilities designate 3 mm as an inspection threshold, our system is specifically designed to achieve a higher-resolution detection capability for cracks as narrow as 1 mm. This approach ensures reliable detection with a sufficient safety margin and enables the proactive monitoring of crack progression for preventive maintenance. The effectiveness of the proposed image processing techniques was validated, with an *F*_1_ score-based analysis revealing key trade-offs between maximizing detection recall and achieving a balanced performance depending on the chosen simulated altitude. Furthermore, evaluation using real-world inspection data demonstrated that the proposed system achieves a detection performance comparable to that of a well-established commercial system, confirming its practical applicability. Crucially, by mapping the detected cracks to real-world coordinates on georeferenced orthophotos, the system provides a foundation for advanced, data-driven asset management, allowing for the quantitative tracking of deterioration over time. These results confirm that the proposed workflow is a practical and sustainable solution for infrastructure monitoring.

## 1. Introduction

Many infrastructure structures in Japan were constructed during the period of rapid economic growth in the 1950s to 1970s. As a result, a significant number of these structures have already exceeded their 50-year design service life, and infrastructure deterioration has become a serious societal issue. According to a report by the Ministry of Land, Infrastructure, Transport and Tourism (MLIT) of Japan, approximately 43% of port facilities will be more than 50 years old by the year 2030, highlighting the urgent need for strategic countermeasures [1].

In order to safely maintain such aging infrastructure, regular inspections are essential. However, current inspection operations rely heavily on manual labor and visual observation, and the shortage of skilled engineers who can perform these inspections appropriately is a growing concern. In particular, port facilities are exposed to harsh salt-laden environments due to seawater, which accelerates the rate of deterioration. Furthermore, these facilities, such as quay walls and coastal levees, often span wide areas, making the introduction of efficient maintenance methods that utilize new technologies a critical and urgent need.

The problem of infrastructure deterioration is not limited to developed nations such as Japan. Many developing countries are beginning to face similar challenges, as infrastructure built in previous decades begins to age without sufficient investment in maintenance and renewal. Gurara et al. [2] highlighted that developing countries, especially those in Asia and Africa, face substantial risks from aging infrastructure due to inadequate resources and lack of regular maintenance protocols. This underscores the global importance of developing sustainable, accessible, and cost-effective infrastructure inspection methods.

To address these global challenges, recent studies have explored the use of advanced technologies such as drones equipped with high-resolution cameras and deep learning techniques for infrastructure inspection. However, existing research primarily utilizes expensive industrial drones and high-cost sensors, which poses significant barriers for their widespread adoption, especially by local governments and developing regions with limited financial and technical resources.

In contrast, this study introduces a cost-effective and accessible method specifically designed for practical implementation by local government personnel, highlighting its suitability for both developed and developing regions. By combining publicly available datasets with domain-specific training data and employing a YOLO (You Only Look Once)-based [3] object detection algorithm, the proposed system achieves high accuracy in detecting cracks as narrow as 1 mm, despite using low-cost cameras and a small general-purpose drone. This approach not only enhances the feasibility of regular and widespread inspections but also contributes significantly to the development of sustainable infrastructure management strategies globally, including regions with resource constraints.

## 2. Related Work

Accurate crack detection is crucial for ensuring the safety and durability of concrete infrastructure. Cracks, which can occur due to environmental factors, material aging, and load conditions, indicate the early stages of structural deterioration. If left unaddressed, they can lead to severe consequences such as rebar corrosion, spalling, and ultimately, structural collapse. Conventional crack detection has relied on manual on-site inspections, which are inefficient, subjective, and insufficient for large-scale maintenance needs. Furthermore, such inspections can be time-consuming, costly, and hazardous for inspectors.

The application of deep learning technologies has become an indispensable approach in the field of crack detection for infrastructure. Recent research trends broadly follow two directions. One direction focuses on improving the architecture of object detection models, such as the YOLO series, aiming for both detection speed and accuracy [4,5,6]. The other direction leverages models such as Vision Transformers to achieve highly precise “semantic segmentation,” identifying crack regions at the pixel level. These advanced studies have achieved remarkable results in optimizing model internals and quantifying fine cracks at the 0.1 mm scale for structures such as bridges, dams, and tunnels [7,8,9,10]. However, while these studies pursue cutting-edge technologies, they often utilize data captured by expensive industrial drones equipped with high-performance cameras [11] and require complex manual parameter tuning or high-performance computing resources [12,13]. This presents practical challenges for local governments responsible for infrastructure maintenance with limited budgets and personnel, as adoption and operation hurdles remain high.

In contrast, this study takes a different approach, aiming to solve real-world operational challenges. Our primary goal is not the pursuit of peak technical performance, but rather the development of an inspection system that can be “sustainably utilized” by local government staff in their daily operations. To achieve this, our study is distinguished from previous research in three key aspects.

First, our study clarifies its target and required specifications. We focus specifically on “port quay walls.” According to the “Inspection and Diagnostic Guidelines for Port Facilities (Part II: Implementation Procedure)” published by Japan’s Ministry of Land, Infrastructure, Transport and Tourism [14], a crack width of 3 mm is set as one inspection threshold. However, it is widely recognized in the field of structural health monitoring that detecting cracks in their early, finer stages is of great importance, as it can prevent more severe damage from occurring [15]. Based on this principle of early detection and to ensure a sufficient safety margin for reliably identifying cracks that approach the 3 mm threshold, this study sets a practical performance target of detecting 1 mm wide cracks.

Second, we employ a unique methodology. Rather than concentrating on complex architectural modifications of state-of-the-art models, we strategically combine three core elements:

- Accessible Equipment: The system is designed to use compact, general-purpose drones (e.g., the Autel Robotics EVO II Pro) that are feasible for local governments to introduce.
- A Proven Detection Model: We adopt YOLOR (You Only Learn One Representation) [16], which has a strong track record of high performance and efficiency, as the basis for object detection.
- Practical Preprocessing Techniques: To compensate for the resolution limits of low-cost cameras, we apply original image processing techniques—”overlapping tile division” and “pseudo-altitude slicing”—to the orthophotos to enhance the detection capabilities.

Third, our contribution differs in its focus. While many previous studies aimed for “high-precision, pixel-level segmentation,” our research emphasizes mapping detected cracks to “real-world coordinates” by utilizing the geospatial information within orthophotos. This enables not only crack detection but also the tracking of temporal changes and quantitative asset management through integration with GIS, paving the way for more advanced infrastructure maintenance. Tsaimou et al.’s study [17], similar to ours, combines drone-derived orthophotos and GIS tools; however, our research distinguishes itself by using a compact general-purpose drone and YOLOR as the foundation, setting a concrete target of 1 mm crack detection and presenting a clear vision for sustainable operation by local governments, thereby offering a more practical solution.

## 3. Proposed Method

A key technical challenge in drone-based crack inspection lies in the limited resolution of onboard imaging sensors, particularly those integrated into compact, low-cost drone platforms widely adopted by local governments. These sensors typically produce Ground Sample Distances (GSDs) of several millimeters per pixel at safe operating altitudes, making it difficult to reliably detect sub-centimeter cracks. For example, aerial imagery captured at a 10 m altitude using the Autel Robotics EVO II Pro yields a GSD of approximately 2.28 mm/pixel (px). While this resolution marginally meets the 3 mm inspection threshold for port quay walls, reliably detecting 1 mm cracks requires additional image processing strategies. To address this issue, we introduce two lightweight preprocessing techniques (overlapping image tiling and pseudo-altitude slicing) that enhance crack detectability without requiring sensor or hardware modifications.

To overcome the technical limitations of image resolution imposed by these low-cost onboard cameras, the proposed system combines deep learning-based image recognition with multiple image processing techniques. This approach enables a crack detection performance that satisfies the inspection standards for port quay walls. In this study, we define “small general-purpose drones” as compact, commercially available drones commonly used by local governments, such as the DJI Mavic 3 (DJI, Shenzhen, China). Our experiments used the Autel Robotics EVO II Pro (Autel Robotics, Shenzhen, China), which offers comparable performance. This drone, measuring 424 × 354 × 110 mm, is equipped with a built-in camera (sensor width: 13.2 mm, focal length: 10.57 mm). Aerial imagery captured at a 10 m altitude (image width: 5472 px) achieved a GSD of approximately 2.28 mm/px, as calculated by Equation (1). The GSD of the DJI Mavic 3 under the same conditions is approximately 2.66 mm/px.

(1) $$Ground\;Sample\;Distance\left[mm/px\right]=\frac{Flight\;altitude\left[mm\right]\times Sensor\;width\left[mm\right]}{Focal\;length\left[mm\right]\times Image\;width\left[px\right]}$$

**Figure 1:**
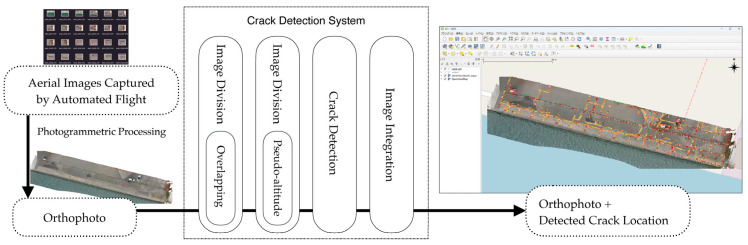
Flowchart of the crack detection system.

Given this resolution, cracks of approximately 3 mm in width, serving as the inspection criteria benchmark for port quay walls, can theoretically be identified from aerial images taken at a 10 m altitude. However, to ensure the reliable identification of 3 mm cracks under diverse field conditions (a key safety margin) and to enable proactive monitoring of deterioration, the system requires the capability to detect cracks around 1 mm in width. Therefore, this study set a target detection width of 1 mm to develop a model with sufficient diagnostic capability for strategic maintenance. Furthermore, from a system operation perspective, merely displaying the detected cracks on individual aerial photographs without knowing their real-world coordinates poses significant limitations for improving maintenance management efficiency. To overcome this issue, our system design utilizes orthophotos, enabling the detected crack locations to be mapped onto real-world coordinates. Figure 1 provides an overview of the proposed crack detection system. The detailed functions corresponding to each step (e.g., image acquisition, orthophoto generation, image division, deep learning-based detection, and result export) will be described in the following sections.

### 3.1. Automatic Measurement Using a Small General-Purpose Drone and Photogrammetric Processing

The inspection of port quay walls is often hindered by challenging site conditions, including elevation changes, sloped surfaces, and the absence of scaffolding. To improve both efficiency and safety in these environments, small general-purpose drones are increasingly being employed for maintenance inspections. The automatic flight and image capture capabilities of these drones allow for consistent, high-quality aerial imagery, regardless of the operator’s skill level, thereby facilitating more efficient and comprehensive inspections of extensive quay walls.

In this study, automatic measurement using a small general-purpose drone was employed to capture aerial images of quay walls at a flight altitude of 10 m. During flight, an overlap rate of approximately 80% was configured. This high degree of overlap is essential for the photogrammetry software to identify sufficient common features between adjacent images, which is critical for robust photo alignment and the accurate reconstruction of a site’s 3D model. Ground Control Points (GCPs) were also established, and Global Navigation Satellite System (GNSS) surveying was conducted, with the placement of aerial targets to improve the positional accuracy of the orthophotos to the centimeter level. While the drone’s onboard GNSS typically provides only meter-level accuracy, the use of survey-grade GCPs is crucial for achieving the centimeter-level absolute accuracy required for reliable infrastructure monitoring and change detection. To ensure high positional accuracy, a total of five GCPs were established, with one placed at each of the four corners, and one in the center of the survey area. In addition to these GCPs, two independent checkpoints were used to validate the final spatial accuracy of the orthophoto. The resulting orthophoto achieved a high degree of accuracy, with a planimetric RMSE of 0.89 cm and a vertical RMSE of 2.74 cm, yielding a total RMSE of 2.88 cm.

The 10 m flight altitude was selected for several reasons: it allows for the identification of cracks measuring a few millimeters, as mentioned in the previous section; flying at lower altitudes would reduce the safety margin relative to nearby structures or buildings; and at lower altitudes, even overlapping photos may lack sufficient common feature points necessary for subsequent photogrammetric processing. In this study, Agisoft Metashape Professional Edition (v. 2.2.0; Agisoft LLC, St. Petersburg, Russia) [18] was used for photogrammetric processing. To prevent visual discontinuities during orthomosaic generation, the “Mosaic” blending mode is typically utilized. This mode automatically calculates optimal seamlines and seamlessly blends overlapping images, preserving detail while creating a continuous result. For specific datasets with significant lighting variations where visual uniformity is prioritized, the “Average” mode may be selected instead. For the sites presented in this study, the “Mosaic” mode provided a sufficiently seamless and detailed result. By leveraging the geographic information embedded in orthophotos, it becomes possible to automatically measure crack dimensions such as length, width, and area and to compare the crack detection results across different time periods to assess changes, which is difficult to accomplish with individual aerial photographs alone.

### 3.2. Image Division Techniques

**Figure 2:**
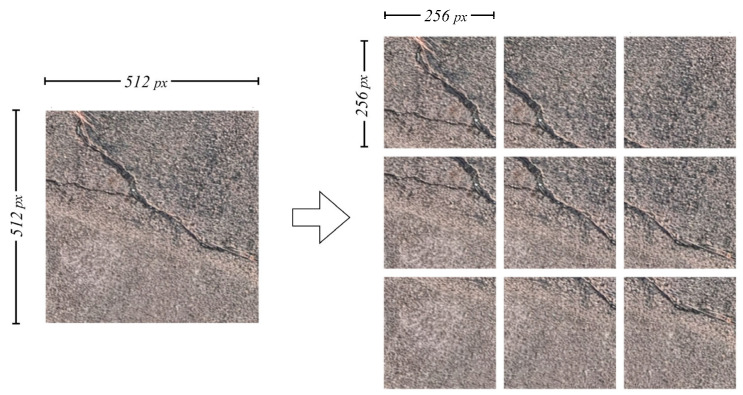
Example of image tiling using the overlapping method (50% overlap).

The orthophotos generated in the previous step were too large in size to be directly used for crack detection (e.g., for a 100 × 20 m site, the image resolution reached 63,747 × 36,319). Therefore, it was necessary to divide these images into appropriately sized tiles. Based on preliminary experiments, it was found that the optimal performance was achieved when the tile size matched the size of the training data used in the deep learning model. Hence, a tile size of 256 × 256 was adopted. To further enhance the detection performance, two division methods were introduced: overlapping image tiling and pseudo-altitude slicing. Both the overlapping tiling and the pseudo-altitude slicing methods were implemented as independent, automated scripts to ensure an efficient and reproducible image division workflow. Overlapping image tiling mitigates the risk of missing cracks that appear along tile boundaries by allowing adjacent tiles to partially overlap. Figure 2 shows an example with a 50% overlap. This technique has been widely used in patch-based deep learning models for crack detection, as it significantly improves the model’s ability to localize cracks near edges [19].

Next, to address the issue where cracks in 256 × 256 tiles differ in scale from the training data, leading to reduced detection performance, a pseudo-altitude slicing method was developed. While prior studies have explored multiscale feature fusion approaches within neural network architectures to improve crack detection robustness [20,21], this study introduces a data preprocessing method that achieves similar multiscale representation without requiring network-level modifications. This technique simulates images captured at different flight altitudes by adjusting the scale of cracks within each tile. Similar in spirit to multiscale feature fusion techniques proposed in previous studies [22], this method enhances the model’s ability to generalize across varying crack scales. In this method, the tile size is recalculated using Equation (2), which incorporates the GSD:

(2) $$Pseudo\;altitude\;tile\;size\left[px\right]=\frac{GSD\;at\;desired\;altitude\;\left[mm/px\right]\times Tile\;size\;at\;original\;altitude\left[px\right]}{GSD\;at\;original\;altitude\left[mm/px\right]}$$

For example, using orthophotos generated from aerial images captured at 10 m altitude, the tile size can be changed from 256 × 256 to 128 × 128 to replicate the crack scale observed at 5 m altitude. In the case of the EVO II Pro used in this study, the GSD at 5 m altitude was 1.14 mm/px, the original tile size was 256, and the GSD at 10 m altitude was 2.28 mm/px. Using Equation (2), the calculated tile size for simulating 5 m altitude became 128. Since all tiles, regardless of their pseudo-altitude scale, were derived from the same source orthophoto, they were inherently georeferenced and perfectly aligned with each other. No additional matching or alignment process was required.

**Figure 3:**
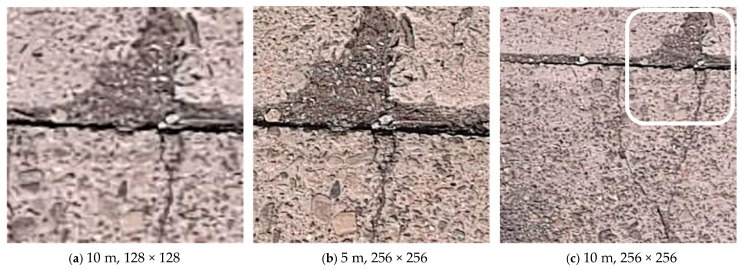
Orthophoto tiles generated from the same location under different tile size configurations: (**a**) pseudo-altitude 5 m (generated from 10 m flight data, 128 × 128); (**b**) actual altitude 5 m, 256 × 256; (**c**) actual altitude 10 m, 256 × 256.

Figure 3 illustrates the effectiveness of the pseudo-altitude slicing method. Image (a) simulates a 5 m altitude observation using a 128 × 128 tile derived from the orthophoto generated from the 10 m altitude flight, while image (b) is an actual 5 m altitude capture at 256 × 256. Although the image sharpness differs, the proportion of crack area within these images is similar, confirming the effectiveness of the scale adjustment through pseudo-altitude slicing. In contrast, image (c), derived directly from the orthophoto generated from the 10 m altitude flight without scale adjustment (256 × 256), exhibits a significantly different crack scale, highlighting the necessity of scale alignment for effective crack detection. The detailed quantitative evaluation of these image division techniques is presented in Section 4.

### 3.3. Crack Detection Model Using Deep Learning

In deep learning, constructing a high-accuracy detection model requires a large amount of training data. However, relying solely on the original dataset prepared in this study (294 image sets) was insufficient for training a robust model. Therefore, we additionally utilized the publicly available dataset SDNET2018 [23] to supplement the training data. Previous literature reviews, such as that by Kaveh and Alhajj [24], have emphasized that combining publicly available datasets with additional, task-specific data significantly enhances a model’s generalization capabilities and practical applicability in diverse structural inspection contexts.

SDNET2018 is an image dataset intended for the training, validation, and benchmarking of AI algorithms for concrete crack detection. The SDNET2018 dataset includes 56,092 images, each with a resolution of 256 × 256, depicting concrete bridge decks, walls, and pavements with and without cracks. The dataset contains cracks ranging in width from 0.06 mm to 25 mm and also includes various image conditions such as shadows, surface roughness, scaling, edges, holes, and background debris. SDNET2018 has been widely adopted in recent studies for training and evaluating concrete crack detection models. For instance, Fang et al. [25] utilized SDNET2018 to develop a hybrid approach combining deep belief networks and an improved fish swarm optimization algorithm, demonstrating its applicability for optimizing model performance under various crack conditions.

In this study, 2300 images resembling cracks typically found on port quay walls were manually selected from the SDNET2018 dataset and annotated with bounding boxes using the open-source annotation tool LabelMe [26] to form an extended dataset for training. The use of SDNET2018 not only increased the volume of training data but also enabled the crack detection model to handle various types of cracks not present in the original dataset, thereby improving the model’s generalization capability. The evaluation of the prepared dataset is discussed in detail in Section 4.

Semantic segmentation approaches, such as U-Net [27], are effective in capturing detailed spatial patterns of cracks but typically require considerable computational resources and longer inference times. In contrast, this study adopted a YOLO-based object detection approach. Specifically, YOLOR achieves a favorable trade-off between detection accuracy, inference speed, and computational efficiency, while maintaining high adaptability to small, custom datasets. Its unique design, integrating explicit and implicit knowledge representations, has been shown to stabilize learning and enhance performance when training data are limited or highly domain-specific.

A primary reason for selecting YOLOR in this study was its built-in genetic algorithm (GA)-based hyperparameter evolution function. This feature enables the automatic tuning of critical parameters such as learning rate, momentum, anchor settings, and data augmentation rates whenever the training dataset is updated or expanded. As a result, continuous model improvement becomes highly practical, especially in real-world scenarios where new data is frequently collected and incorporated. In contrast, state-of-the-art models such as YOLO11 [28] do not natively support GA-based hyperparameter optimization in their official implementations, which limits the ease and efficiency of large-scale or continuous parameter tuning when adapting the model to newly acquired or incrementally expanded datasets. This practical advantage makes YOLOR particularly suitable for iterative training and ongoing inspection system improvement in infrastructure management.

The crack detection model was trained using the YOLOR-D6 architecture (yolor-d6.yaml) with no modifications to the network structure or attention modules. The input image size was set to 256 × 256, with a batch size of 128 and 2000 training epochs. Key hyperparameters such as learning rate, momentum, weight decay, and anchor boxes were automatically tuned using a GA to optimize the performance on the validation set, rather than using default or manually selected values (Table 1). This approach allowed for robust model convergence and improved generalization compared to manual hyperparameter selection. The inference speed was 26.87 FPS, evaluated on an NVIDIA RTX 3090 GPU (NVIDIA Corporation, Santa Clara, CA, USA). The model processed 256 × 256 tiles at a batch size of 128.

The practical effectiveness of YOLOR in detection tasks has been verified by previous research. For example, Guzmán-Torres et al. [29] successfully applied YOLOR for real-time vehicle detection and tracking in urban traffic environments, confirming its robustness under diverse real-world conditions.

**Figure 4:**
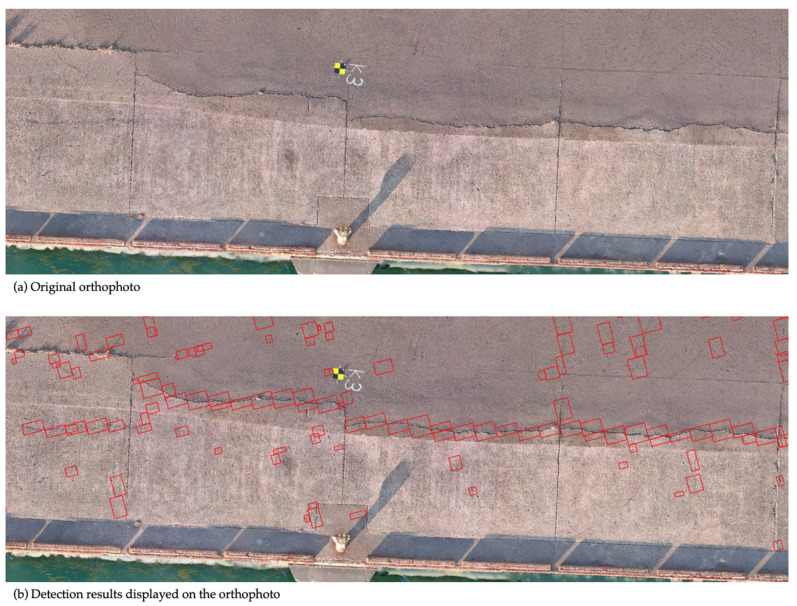
(**a**) Original orthophoto and (**b**) detection results displayed on the orthophoto. Red boxes: cracks detected by the system.

In the developed crack detection process, orthophoto tiles obtained through the image division methods described in Section 3.2 are individually analyzed to identify and localize cracks. The deep learning model was implemented using the PyTorch (v. 1.7.1; Meta AI, New York, NY, USA) [30] framework, and the detected cracks were visualized as bounding boxes (Figure 4). To facilitate practical inspection management, the system assigns geographic coordinates calculated from orthophotos to each detected crack and outputs this information in JavaScript Object Notation (JSON) format. This structured data enables tracking crack progression over time by comparing the inspection results across multiple periods using GIS. Additionally, this method lays the groundwork for future enhancements, such as automatically calculating the total length of cracks and assessing the structural health of each inspection unit, thus contributing to the quantitative evaluation of port quay wall conditions.

The integration of geographic information with AI-based detection results significantly improves the capabilities for spatiotemporal analysis and infrastructure management. Similar benefits have been demonstrated in recent geospatial AI studies that mapped risks such as flood exposure in national road networks, highlighting the broader applicability and utility of location-aware detection systems [31].

## 4. Results

In this section, we first evaluate the effectiveness of the training datasets and image division techniques prepared in this study. Based on these evaluations, we identify the dataset and division strategy that deliver the highest detection performance. Next, to assess the practical applicability of the developed system, we conduct a comparative analysis against a well-established commercial system. This involves comparing the results from conventional visual inspections with those obtained from the detection system to determine how accurately the system can detect cracks with a target width of 1 mm, which is the main focus of this study.

As evaluation metrics, we used *Precision*, which indicates the accuracy of the detected results, and *Recall*, which measures how comprehensively cracks are detected. *Precision* and *Recall* were calculated using Equations (3) and (4), respectively, and ground truth crack labels prepared based on visual inspection.

(3) $$Precision=\frac{Number\;of\;detected\;boxes\;containing\;cracks}{Total\;number\;of\;detected\;boxes}\times 100$$

(4) $$Recall=\frac{Number\;of\;crack\;pixels\;within\;detected\;boxes}{Total\;number\;of\;crack\;pixels}\times 100$$

For the evaluation of the training datasets and the overlapping image tiling method, aerial images captured at flight altitudes of 5 m, 10 m, 15 m, and 20 m were used to assess the influence of flight altitude on detection performance. For evaluating the pseudo-altitude slicing method, orthophotos generated from aerial images taken at a flight altitude of 10 m were utilized.

### 4.1. Comparative Evaluation Using Different Datasets

**Figure 5:**
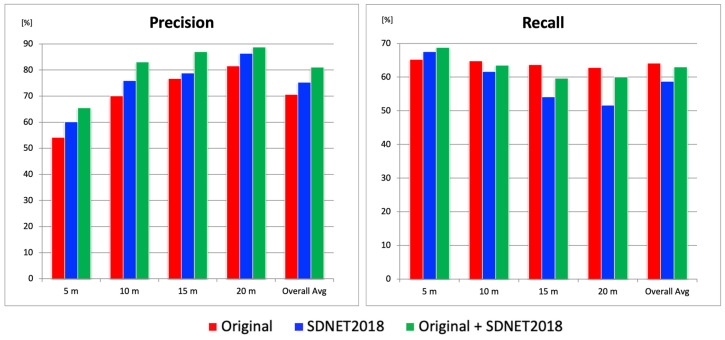
*Precision* and *Recall* results for each dataset.

In this study, two types of training datasets were used for YOLOR model training: an original dataset (294 image sets) [32] created from aerial images obtained independently, and the publicly available dataset SDNET2018 (2300 image sets). In this section, three types of object detection models were trained using (1) the original dataset only; (2) SDNET2018 only; (3) a combination of both datasets. We thus determined which dataset configuration yielded the best performance. Since deep learning inference results may vary depending on the model, five models were trained for each dataset configuration. For each model, *Precision* and *Recall* were calculated and then averaged. The results are shown in Figure 5.

For evaluation, aerial images captured at altitudes of 5 m, 10 m, 15 m, and 20 m at three test sites were used. The *Precision* results showed that, across all altitudes and in overall averages, the combination of the original dataset and SDNET2018 yielded the highest scores, while the original dataset alone produced the lowest scores. The average difference in *Precision* between these two configurations was approximately 10%. On the other hand, in terms of *Recall*, the original + SDNET2018 configuration showed the highest *Recall* at 5 m altitude. The superior *Recall* at 5 m altitude achieved with the original + SDNET2018 configuration was likely due to the abundance of fine crack images in SDNET2018. These images enhanced the model’s sensitivity to micro-cracks, particularly under the higher resolution conditions afforded by low-altitude imagery. In contrast, at other altitudes, the original-only configuration yielded the highest *Recall*. However, the differences in *Recall* were relatively small compared to the *Precision* gap: 1.3% at 10 m, 4.0% at 15 m, 2.7% at 20 m, and 1.1% for the overall average. Although the 4.0% difference at 15 m was somewhat large, the overall average difference in *Recall* remained around 1.1%. Considering the 10% gap in *Precision*, the combination of the original and SDNET2018 datasets was considered the most balanced and optimal for constructing a robust crack detection model, achieving a *Precision* of 81.0% and a *Recall* of 62.9%.

### 4.2. Performance Evaluation of the Overlapping Image Tiling Method

**Figure 6:**
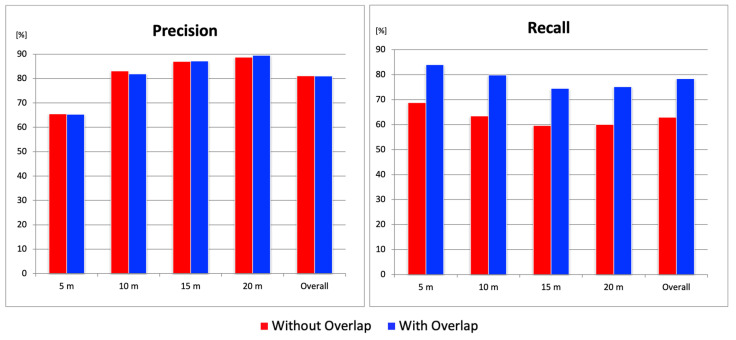
*Precision* and *Recall* results from the overlapping image tiling method.

**Figure 7:**
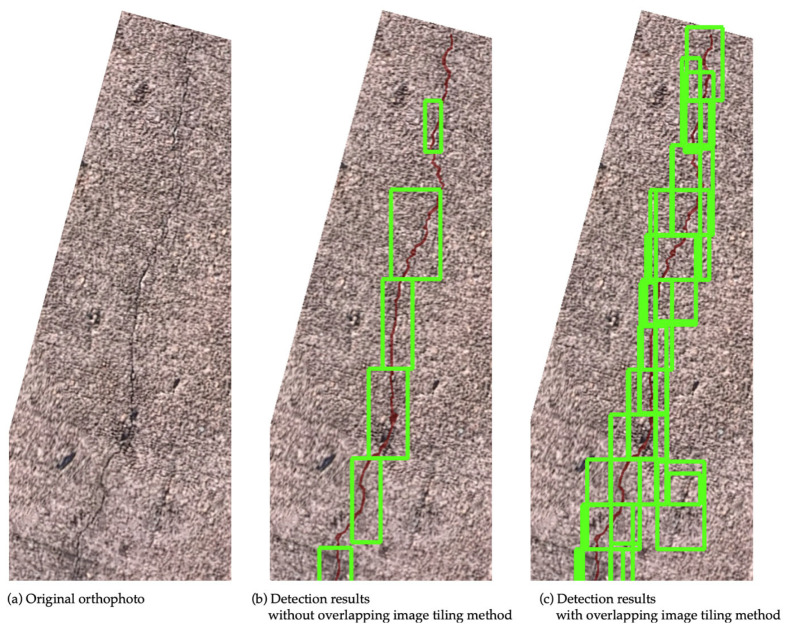
(**a**) Original orthophoto; (**b**) detection results without overlapping image tiling method; (**c**) detection results with overlapping image tiling method. Red regions indicate ground truth cracks; green bounding boxes show crack detections.

This section evaluates the effectiveness of the overlapping image tiling method. Five models trained using the combined original + SDNET2018 datasets (as selected in the previous section) were used to calculate the average *Precision* and *Recall* with and without the overlapping tiling method, in order to verify its effectiveness. In terms of *Precision*, there was a slight decrease of about 1% at a flight altitude of 10 m when overlapping was applied, but no significant improvement was observed. On the other hand, on average, the overlapping method increased *Recall* by approximately 24% relative to the non-overlapping condition, quantitatively demonstrating the effectiveness of the method. Notably, at an altitude of 5 m, *Recall* improved to over 80% (Figure 6). Comparing the detection results with and without overlapping, cracks that had previously been missed without overlapping were successfully detected when overlapping was introduced (Figure 7). A detailed look at the divided image tiles where missed detections occurred showed that the cracks were located at the boundaries of the images. Through the use of overlapping tiles, these cracks were brought closer to the center of the tile, which allowed them to be detected successfully. From these results, it can be concluded that the overlapping image tiling method significantly reduced the missed detections caused by tile boundaries and provided stable detection performance regardless of flight altitude.

### 4.3. Performance Evaluation of the Pseudo-Altitude Slicing Method

In this section, we evaluate the pseudo-altitude slicing method, which adjusts the scale of cracks within tiled images to improve *Recall*. For this verification, orthophotos of port quay walls generated from aerial images captured at an actual flight altitude of 10 m were cropped at the test sites, and *Precision*, *Recall*, and *F*_1_ score were calculated. Based on the actual flight altitude of 10 m, pseudo-altitudes of 5 m and 15 m were simulated. Four combinations were evaluated, as follows: (1) 10 m only; (2) pseudo 5 m + 10 m; (3) 10 m + pseudo 15 m; (4) pseudo 5 m + 10 m + pseudo 15 m. The tile sizes were set to 128 for pseudo 5 m, 256 for 10 m, and 384 for pseudo 15 m. Each tile size was calculated using Equation (2) based on the corresponding pseudo-altitude setting.

**Figure 8:**
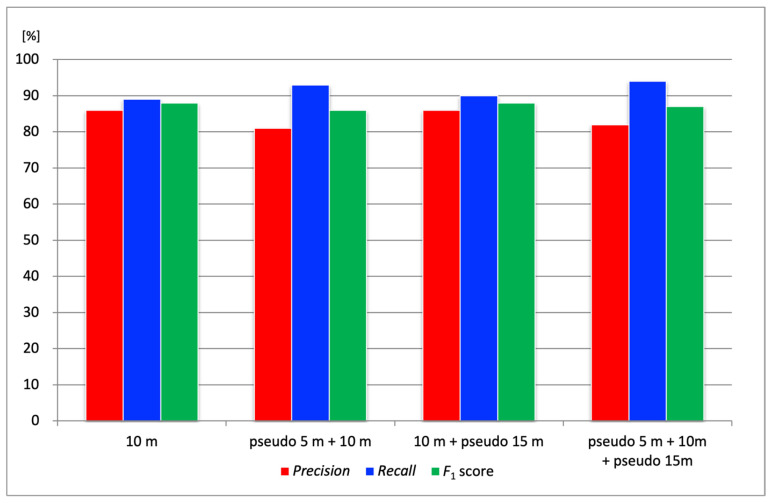
Results of the pseudo-altitude slicing method. Red bars represent *Precision*, blue bars represent *Recall*, and green bars represent *F*_1_ score at different tile configurations.

Figure 8 shows the *Precision*, *Recall*, and *F*_1_ score results for the four altitude combinations at three test sites. The *F*_1_ score, which represents the harmonic mean of *Precision* and *Recall*, was calculated using Equation (5):

(5) $$F_{1}=\frac{2\cdot \mathrm{Precision}\cdot \mathrm{Recall}}{\mathrm{Precision}+\mathrm{Recall}}$$

**Figure 9:**
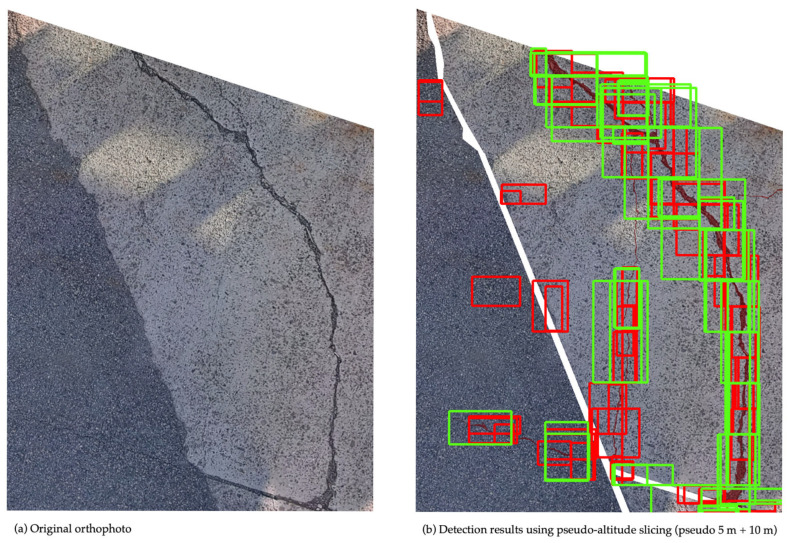
(**a**) Original orthophoto and (**b**) detection results using pseudo-altitude slicing. Red regions indicate ground truth cracks; white areas are masked out. Red bounding boxes show crack detections from the pseudo 5 m tiles, and green bounding boxes show detections from the 10 m tiles.

In terms of *Precision*, the combination of pseudo-altitudes produced varied results depending on the test site, and no consistent improvement was observed. However, *Recall* tended to improve overall when combining pseudo-altitudes. For example, Figure 9 illustrates a crack (highlighted in red) that was not detected in the aerial image at 10 m altitude (detected area shown as a green bounding box) but was successfully detected using the pseudo 5 m tiling approach (detected area shown as a red bounding box). To ensure a fair comparison, concrete joints, which are not actual cracks, were excluded from the evaluation by applying masks. The white-shaded areas in the figure indicate these masked regions. This result suggests that the crack could not be detected in the 10 m image due to a mismatch in scale with the training data, whereas adjusting the tile size using the pseudo-altitude slicing method enabled successful detection. Compared to using 10 m images alone, the combination with pseudo 5 m images improved the overall average *Recall* by approximately 4%. This improvement was attributed to the pseudo-altitude slicing method making it easier for the crack scale in the images to match that of the training data. In particular, the ability to detect previously missed narrow cracks is expected to contribute significantly to preventing oversights in practical applications.

While all combinations using pseudo-altitudes showed improved *Recall*, the *F*_1_ score provides a more balanced assessment of overall performance. The more rigorous analysis, shown in Figure 8, revealed that the “10 m + pseudo 15 m” combination achieved the highest *F*_1_ score (88%), demonstrating the best trade-off between *Precision* and *Recall*. This suggests that while the “pseudo 5 m” setting was effective for maximizing *Recall*, the simulation of a higher altitude (“pseudo 15 m”) contributed to a better overall balanced performance. Therefore, the optimal configuration depends on the inspection objective. The “10 m + pseudo 15 m” combination is recommended when prioritizing balanced performance, while a configuration including “pseudo 5 m” is considered suitable if the primary objective is to prevent missed detections, a key focus of this study.

### 4.4. Benchmark Comparison with a Commercial System

**Figure 10:**
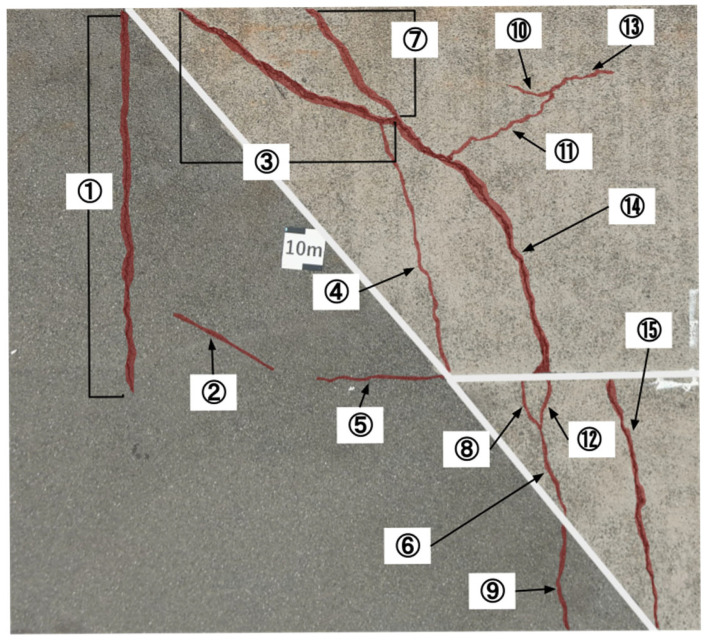
Labeled image of cracks at the test site. Red: ground truth cracks; white: masked areas; numbers denote crack IDs.

In the previous sections, the effectiveness of the developed image division techniques and datasets was evaluated using the performance metrics defined in this study, namely, *Precision* and *Recall*. In this section, we benchmarked the crack detection system developed in this study against a well-established commercial system to assess its current level of practicality. The benchmark evaluation was conducted at the same three test sites as before. At each site, cracks of varying widths, recorded during visual inspection, were each given a unique crack ID number for tracking and analysis. The presence or absence of detection for each crack was compared between the two systems. Note that the performance metrics from previous sections were not used here because our system outputs detected cracks as rectangular bounding boxes, whereas the commercial system visualizes crack areas by coloring them. Due to this fundamental difference in output format, the metrics used earlier were not suitable for direct comparison in this benchmark evaluation. As for the results from the three test sites, the developed system showed more false positives due to vegetation and other noise, but the crack detection rate was comparable to that of the commercial system, confirming near-equal performance. For instance, at one of the test sites shown in Figure 10, differences were observed in the detection results for cracks #4 (width 1 mm), #9 (width over 5 mm), and #13 (width 0.5 mm), as shown in Table 2. Cracks #4 and #9 were partially missed by our system but fully detected by the commercial system. On the other hand, crack #13 was partially detected by our system but missed entirely by the commercial system. These results demonstrate that our system is capable of detecting certain cracks that the commercial system fails to detect. Moreover, all cracks with a width of 1 mm, excluding #4, were successfully detected, indicating that the developed system meets the target detection performance for 1 mm cracks set in this study. Further analysis of false positives and missed detections revealed that the primary sources of errors were vegetation and minor debris. Introducing a secondary classification stage to filter these false detections is expected to substantially improve overall *Precision*, as discussed in Section 5.

From a system perspective beyond detection accuracy, the workflow of the commercial system is similar to the one that our study aimed to achieve. However, being a commercial product, its technical specifications are not publicly available. Moreover, it requires high-performance and costly image acquisition equipment to guarantee its performance. It also imposes limitations on image size and the number of images that can be processed. Additionally, the detection results produced by the commercial system do not contain geographic information, making it difficult to apply the results in practical scenarios. In contrast, our system leverages open-source tools, and many implementation details and use cases are publicly available. There are no restrictions on image size or quantity, and the output includes geographic coordinates, enabling easy time-series analysis and integration with other geospatial data for advanced analysis. These aspects demonstrate the advantages of our system over commercial alternatives.

## 5. Conclusions

This study aimed to develop a sustainable inspection method for port infrastructure, providing a practical alternative to conventional approaches that often rely on expensive, specialized equipment. By combining deep learning technologies with advanced image division techniques, the system successfully utilized a small general-purpose drone to achieve its target of detecting cracks as narrow as 1 mm. This capability is critical not only for meeting the inspection criteria for port quay walls but also for enabling proactive maintenance through the early detection and monitoring of structural deterioration. A key contribution of this work is the integration of these detections with georeferenced orthophotos, establishing a workflow that supports quantitative, data-driven asset management. While many studies have focused on optimizing the detection metrics of YOLO models, our contribution lies in demonstrating a holistic system that is specifically designed for accessibility, low-cost operation, and integration with geospatial asset management workflows. Experimentally, the system’s effectiveness was validated through several key findings. Training on a combination of public (SDNET2018) and original datasets proved most effective for creating a robust model. The proposed image division techniques were also crucial for performance: the overlapping tiling method significantly increased *Recall* by approximately 24%, while the pseudo-altitude slicing method offered a nuanced trade-off, with the *F*_1_ score-based analysis revealing that a combination of 10 m and “pseudo 15 m” data provided the best-balanced performance.

To evaluate the practicality of the developed crack detection system, a performance comparison was conducted with a commercial system. Although the proposed system demonstrated comparable detection rates, it revealed a challenge in reduced *Precision* due to false positives, which were primarily caused by vegetation, accumulated debris, or stains being mistakenly detected as cracks. To mitigate this issue, future work will incorporate a two-stage detection approach by adding a classification model after initial detection, specifically designed to filter out non-crack objects such as vegetation and debris. Similar two-stage frameworks have proven effective in prior deep learning-based crack detection studies, distinguishing true cracks from non-structural elements such as stains or debris [33,34]. In addition to reducing false positives, future work will also focus on improving the recall rate by addressing false negatives (missed detections), particularly for the very fine cracks that are challenging for low-cost sensors. This will be pursued by enhancing the training dataset with more diverse crack imagery and by exploring the use of super-resolution techniques as a preprocessing step to digitally enhance the visibility of fine cracks.

To enhance the reproducibility and practical utility of the proposed system, key components of the detection code and processing modules will be publicly released on GitHub. Additionally, the original annotated dataset comprising 294 drone-captured crack images is already available at: https://github.com/omu-geolab/CrackImageDatasetForDeepLearning (accessed on 1 July 2025). These publicly accessible resources are expected to support further research and encourage the adoption of advanced inspection methodologies, particularly by local governments and infrastructure managers in regions with limited technical resources.

Since the current system remains a prototype, the next phase of development will focus on enhancing its practical deployability. A lightweight, platform-independent implementation will be created, featuring a user-friendly, web-based interface designed specifically for on-site inspections. This approach aims to enable non-technical personnel, such as local government staff, to easily upload orthophotos and receive real-time crack detection results without the need for specialized computing resources. Pilot deployments and usability assessments will be conducted in collaboration with municipal agencies to ensure that the system seamlessly integrates into existing inspection workflows and effectively meets the operational needs.

## Figures and Tables

**Table 1:** Structure and key hyperparameters of the YOLOR-D6 model used in this study. Hyperparameters marked as “GA evolution results” were tuned using a genetic algorithm.

Category	Parameter	Value/Description	Source/File
Architecture	Model	YOLOR-D6	yolor-d6.yaml
	Pretrained Weights		yolor-d6.pt
	Input Image Size	256 × 256 px	train.py
	Number of Classes	1	yolor-d6.yaml
Anchors	P3 (stride 8)	[19, 27], [44, 40], [38, 94]	yolor-d6.yaml
	P4 (stride 16)	[96, 68], [86, 152], [180, 137]	yolor-d6.yaml
	P5 (stride 32)	[140, 301], [303, 264], [238, 542]	yolor-d6.yaml
	P6 (stride 64)	[436, 615], [739, 380], [925, 792]	yolor-d6.yaml
Training	Batch Size	128	train.py
	Epochs	2000	train.py
Hyperparameters	Learning Rate (lr0)	0.0141	GA evolution results
	Final LR (lrf)	0.186	GA evolution results
	Momentum	0.98	GA evolution results
	Weight Decay	0.00071	GA evolution results
	Anchor T (anchor_t)	4.57	GA evolution results
	Warmup Epochs	1.9	GA evolution results
	Warmup Momentum	0.95	GA evolution results
	Box Loss Gain	0.02	GA evolution results
	Class Loss Gain	0.51	GA evolution results
	Object Loss Gain	0.604	GA evolution results
	IoU Threshold	0.2	GA evolution results
	Data Augmentation	fliplr: 0.5, mosaic: 0.905, translate: 0.356, scale: 0.418, …	GA evolution results
Other	Optimizer	Stochastic Gradient Descent	train.py/default

**Table 2:** Comparison of the detection results at the test site. ◯: Fully detected; △: partially detected; ✕: not detected.

ID	Crack Width [mm]	Proposed System	Commercial System
①	≥5	◯	◯
②	≥5	△	△
③	≥5	◯	◯
④	1	△	◯
⑤	2	△	△
⑥	≥5	◯	◯
⑦	3	◯	◯
⑧	1	◯	◯
⑨	≥5	△	◯
⑩	0.4	◯	◯
⑪	0.8	△	△
⑫	2	◯	◯
⑬	0.5	△	**✕**
⑭	≥5	◯	◯
⑮	≥5	◯	◯

## Data Availability

The original data of the crack image dataset for deep learning presented in the study are openly available in GitHub at [https://github.com/omu-geolab/CrackImageDatasetForDeepLearning (accessed on 1 July 2025)].

## Abbreviations

The following abbreviations are used in this manuscript:

CNN	Convolutional Neural Network
GA	Genetic Algorithm
GCP	Ground Control Point
GIS	Geographic Information System
GNSS	Global Navigation Satellite System
GSD	Ground Sample Distance
IoU	Intersection over Union
JSON	JavaScript Object Notation
MLIT	Ministry of Land, Infrastructure, Transport and Tourism
YOLO	You Only Look Once
YOLOR	You Only Learn One Representation

## References

[1] Ministry of Land, Infrastructure, Transport and Tourism (MLIT) Current Status and Future of Aging Infrastructure. https://www.mlit.go.jp/sogoseisaku/maintenance/02research/02_01.html.

[2] Gurara D., Klyuev V., Mwase N., Presbitero A., Xu X.C. (2017). Trends and Challenges in Infrastructure Investment in Low-Income Developing Countries. IMF Working Paper. https://www.imf.org/en/Publications/WP/Issues/2017/11/07/Trends-and-Challenges-in-Infrastructure-Investment-in-Low-Income-Developing-Countries-45339.

[3] Redmon J., Divvala S., Girshick R., Farhadi A. (2016). You Only Look Once: Unified, Real-Time Object Detection. arXiv.

[4] Dong X., Liu Y., Dai J. (2024). Concrete Surface Crack Detection Algorithm Based on Improved YOLOv8. Sensors.

[5] Li Q., Zhang G., Yang P. (2024). CL-YOLOv8: Crack Detection Algorithm for Fair-Faced Walls Based on Deep Learning. Appl. Sci..

[6] Sohaib M., Arif M., Kim J.-M. (2024). Evaluating YOLO Models for Efficient Crack Detection in Concrete Structures Using Transfer Learning. Buildings.

[7] Liu G., Wu X., Dai F., Liu G., Li L., Huang B. (2025). Crack-MsCGA: A Deep Learning Network with Multi-Scale Attention for Pavement Crack Detection. Sensors.

[8] Zhang H., Ma L., Yuan Z., Liu H. (2024). Enhanced Concrete Crack Detection and Proactive Safety Warning Based on I-ST-UNet Model. Autom. Constr..

[9] Zhou J., Zhao G., Li Y. (2024). Vison Transformer-Based Automatic Crack Detection on Dam Surface. Water.

[10] Chen S., Feng Z., Xiao G., Chen X., Gao C., Zhao M., Yu H. (2024). Pavement Crack Detection Based on the Improved Swin-Unet Model. Buildings.

[11] Ding W., Yang H., Yu K., Shu J. (2023). Crack Detection and Quantification for Concrete Structures Using UAV and Transformer. Autom. Constr..

[12] Ye G., Dai W., Tao J., Qu J., Zhu L., Jin Q. (2024). An Improved Transformer-Based Concrete Crack Classification Method. Sci. Rep..

[13] Shahin M., Chen F.F., Maghanaki M., Hosseinzadeh A., Zand N., Khodadadi Koodiani H. (2024). Improving the Concrete Crack Detection Process via a Hybrid Visual Transformer Algorithm. Sensors.

[14] Ministry of Land, Infrastructure, Transport and Tourism (MLIT) (2014). Inspection and Diagnosis Guidelines for Port Facilities [Part 2: Implementation Manual]. https://www.mlit.go.jp/kowan/content/001734486.pdf.

[15] Roy S., Yogi B., Majumdar R., Ghosh P., Das S.K. (2025). Deep Learning-Based Crack Detection and Prediction for Structural Health Monitoring. Discov. Appl. Sci..

[16] Wang C.-Y., Yeh I.-H., Liao H.-Y.M. (2021). You Only Learn One Representation: Unified Network for Multiple Tasks. arXiv.

[17] Tsaimou C.N., Kagkelis G., Sartampakos P., Karantzalos K., Tsoukala V.K. (2024). Mapping Cracks on Port Concrete Pavements by Analyzing Structural Health Monitoring Metadata with Computer Vision-Based Techniques. Complex Eng. Syst..

[18] Agisoft Metashape Professional Edition. https://www.agisoft.com/features/professional-edition/.

[19] Hamishebahar Y., Guan H., So S., Jo J. (2022). A Comprehensive Review of Deep Learning-Based Crack Detection Approaches. Appl. Sci..

[20] Ju X., Zhao X., Qian S. (2022). TransMF: Transformer-Based Multi-Scale Fusion Model for Crack Detection. Mathematics.

[21] Ashraf A., Sophian A., Bawono A.A. (2024). Crack Detection, Classification, and Segmentation on Road Pavement Material Using Multi-Scale Feature Aggregation and Transformer-Based Attention Mechanisms. Constr. Mater..

[22] Yang Y., Niu Z., Su L., Xu W., Wang Y. (2023). Multi-scale Feature Fusion for Pavement Crack Detection Based on Transformer. Math. Biosci. Eng..

[23] Dorafshan S., Thomas R.J., Maguire M. (2018). SDNET2018: An Annotated Image Dataset for Non-Contact Concrete Crack Detection Using Deep Convolutional Neural Networks. Data Brief.

[24] Kaveh A., Alhajj R. (2024). Recent Advances in Crack Detection Technologies for Structures: A Survey of 2022–2023 Literature. Front. Built Environ..

[25] Fang Z., Wang X., Gao J., Eskandarpour B. (2025). Optimizing Concrete Crack Detection: An Echo State Network Approach with Improved Fish Migration Optimization. Sci. Rep..

[26] Russell B.C., Torralba A., Murphy K.P., Freeman W.T. (2008). LabelMe: A Database and Web-Based Tool for Image Annotation. Int. J. Comput. Vis..

[27] Ronneberger O., Fischer P., Brox T., Navab N., Hornegger J., Wells W.M., Frangi A.F. (2015). U-Net: Convolutional Networks for Biomedical Image Segmentation. Lecture Notes in Computer Science, Proceedings of the International Conference on Medical Image Computing and Computer-Assisted Intervention (MICCAI), Munich, Germany, 5–9 October 2015.

[28] Ultralytics YOLO, Version 11. https://docs.ultralytics.com/ja/models/yolo11/.

[29] Guzmán-Torres J.A., Domínguez-Mota F.J., Tinoco-Guerrero G., García-Chiquito M.C., Tinoco-Ruíz J.G. (2024). Efficacy Evaluation of You Only Learn One Representation (YOLOR) Algorithm in Detecting, Tracking, and Counting Vehicular Traffic in Real-World Scenarios, the Case of Morelia México: An Artificial Intelligence Approach. AI.

[30] PyTorch. https://pytorch.org.

[31] Rezvani S.M.H.S., Silva M.J.F., Almeida N.M.d. (2023). Mapping Geospatial AI Flood Risk in National Road Networks. ISPRS Int. J. Geo-Inf..

[32] Yoshida D. (2025). Crack Image Dataset for Deep Learning. https://github.com/omu-geolab/CrackImageDatasetForDeepLearning.

[33] Mishra M., Jain V., Singh S.K., Maity D. (2022). Two-Stage Method Based on the You Only Look Once Framework and Image Segmentation for Crack Detection in Concrete Structures. Arch. Struct. Constr..

[34] Sorilla J., Chu T.S.C., Chua A.Y. (2024). A UAV Based Concrete Crack Detection and Segmentation Using 2-Stage Convolutional Network with Transfer Learning. HighTech Innov. J..

